# *C9orf72* Hexanucleotide Repeat Expansion-Related Neuropathology Is Attenuated by Nasal Rifampicin in Mice

**DOI:** 10.3390/biomedicines10051080

**Published:** 2022-05-06

**Authors:** Yukari Hatanaka, Tomohiro Umeda, Keiko Shigemori, Toshihide Takeuchi, Yoshitaka Nagai, Takami Tomiyama

**Affiliations:** 1 Department of Translational Neuroscience, Osaka City University Graduate School of Medicine, Osaka 545-8585, Japan; m20mc009@yf.osaka-cu.ac.jp (Y.H.); umemaru@med.osaka-cu.ac.jp (T.U.); d20ma003@eb.osaka-cu.ac.jp (K.S.); 2 Department of Neurology, Kindai University Faculty of Medicine, Osakasayama 589-8511, Japan; takeuchi@med.kindai.ac.jp (T.T.); yoshi.nagai@med.kindai.ac.jp (Y.N.); 3 Life Science Research Institute, Kindai University, Osakasayama 589-8511, Japan

**Keywords:** *C9orf72*, frontotemporal dementia (FTD), amyotrophic lateral sclerosis (ALS), hexanucleotide repeat expansion (HRE), RNA foci, double-strand RNA-dependent protein kinase (PKR), repeat-associated non-ATG (RAN) translation, dipeptide repeat protein (DPR), TDP-43, rifampicin

## Abstract

The non-coding GGGGCC hexanucleotide repeat expansion (HRE) in *C9orf72* gene is a dominant cause of frontotemporal dementia (FTD) and amyotrophic lateral sclerosis (ALS). This intronic mutation elicits the formation of nuclear and cytoplasmic inclusions containing RNA, RNA-binding proteins, and HRE-derived dipeptide repeat proteins (DPRs), leading to neurodegeneration via the gain-of-toxic function or loss-of-function of relevant proteins. Using C9-500 mice harboring ~500 repeats of the GGGGCC sequence in human *C9orf72* gene, we investigated the effects of rifampicin against HRE-related pathological phenotypes. Rifampicin was administered intranasally to 4.5- to 5-month-old mice for 1 month, and their cognitive function and neuropathology were assessed by the Morris water maze test and immunohistochemical staining. Rifampicin treatment reduced the formation of RNA foci and cytoplasmic inclusions containing DPRs or phosphorylated TDP-43, and furthermore, the levels of phosphorylated double-strand RNA-dependent protein kinase (PKR) that regulates repeat-associated non-ATG (RAN) translation. Synapse loss in the hippocampus and neuronal loss and microglial activation in the prefrontal and motor cortices were also attenuated, and mouse memory was significantly improved. Our findings suggest a therapeutic potential of nasal rifampicin in the prevention of *C9orf72*-linked neurodegenerative disorders.

## 1. Introduction

The GGGGCC hexanucleotide repeat expansion (HRE) in the *C9orf72* gene is the most common genetic cause of frontotemporal dementia (FTD) and amyotrophic lateral sclerosis (ALS) [1]. In normal individuals, the intronic GGGGCC sequence between two non-coding exons (1a and 1b) is repeated less than 30 times, but in patients, it is repeated 100–5000 times [2]. Three mechanisms have been proposed by which HRE causes disease [3,4]: one is the loss-of-function of C9orf72 protein, and the others are the gain-of-toxic function of HRE-derived RNAs and proteins. In the first mechanism, HRE inhibits the transcription of the *C9orf72* gene, probably by forming the G-quadruplex structure in a promoter region, which acts as a steric block to the transcription machinery [5]. C9orf72 protein is expressed in the brain, spinal cord, and immune cells, and has been shown to regulate autophagy and vesicular trafficking [4]. The reduced expression of C9orf72 causes haploinsufficiency, resulting in autophagy dysfunction [6] and neurodegeneration [7]. In the second mechanism, HRE-derived sense and antisense RNAs form the G-quadruplex and hairpin structures [8,9]. These structures sequester some RNA-binding proteins, such as heterogeneous nuclear ribonucleoprotein (hnRNP) H, into nuclear and occasionally cytoplasmic inclusions, i.e., RNA foci [9,10,11]. This sequestering may cause disturbed RNA processing, leading to the RNA-mediated cytotoxicity [12]. Finally, in the third mechanism, from the HRE-derived sense and antisense RNAs, several dipeptide repeat proteins (DPRs), including poly-GA, poly-GP, poly-GR, poly-AP, and poly-PR, are synthesized by repeat-associated non-ATG (RAN) translation [13,14,15,16,17]. These DPRs self-aggregate to form cytoplasmic and occasionally nuclear inclusions that involve an autophagy- and ubiquitin-proteasome-related protein, p62 [13,14,16,18,19]. In addition, HRE-derived RNAs and DPRs promote aberrant cytoplasmic liquid-liquid phase separation (LLPS) to form membrane-less organelles called stress granules, in which RNA, RNA-binding proteins, such as TDP-43, and translation machinery are condensed [20,21,22]. Disease-related LLPS was first suggested for FUS protein [23,24], but now many proteins with the low-complexity domain (LCD), including TDP-43 and other RNA-binding proteins, have been implicated in this phenomenon [25]. Furthermore, HRE-derived RNAs and DPRs inhibit the nuclear-cytoplasm transport by forming nuclear pore complexes [22,26,27]. These alterations accelerate the cytoplasmic accumulation of TDP-43 and its self-aggregation in stress granules, leading to the formation of cytoplasmic TDP-43 inclusions and the depletion of nuclear TDP-43, which causes loss-of-function of the protein [22,28]. The presence of cytoplasmic inclusions of phosphorylated TDP-43 (pTDP-43) and loss of the nuclear localization of TDP-43 are hallmarks of *C9orf72*-linked FTD/ALS [28]. Furthermore, TDP-43 forms toxic oligomers within cells in the brains of FTD and ALS patients [28,29,30]. TDP-43 inclusions, but not RNA foci or DPR inclusions, appear to be associated with neurodegeneration [2,19], suggesting that TDP-43 is an eventual effector of *C9orf72* HRE mutations.

These studies suggest that inhibiting G-quadruplex formation and RAN translation may prevent *C9orf72*-linked disorders. RAN translation is shown to be highly regulated by the double-strand RNA-dependent protein kinase (PKR), which is activated by disease relevant repeat expansion RNAs via the phosphorylation at Thr446 and Thr451 residues [31]. Furthermore, metformin, a medicine to treat type 2 diabetes, has been reported to suppress RAN translation by inhibiting PKR phosphorylation and improve phenotypes in C9-500 mice [31]. Thus, inhibiting PKR phosphorylation may lead to the deterrence of RAN translation. Previously, we showed that a well-known antibiotic, rifampicin, inhibits the aggregation of amyloidogenic proteins, including Aβ, tau, and α-synuclein, in vitro and improves cognition in mouse models of Alzheimer’s disease (AD), tau-associated FTD, and dementia with Lewy bodies (DLB) [32,33]. These findings suggest that rifampicin may be effective at inhibiting DPR and TDP-43 aggregation. Furthermore, should rifampicin have activity to inhibit the formation of RNA foci, it could be a good candidate for a preventive medicine against *C9orf72*-linked FTD/ALS. Thus, in the present study, we studied the effects of rifampicin on the formation of RNA foci and cytoplasmic inclusions composed of DPRs or TDP-43 in the brains of C9-500 mice. The C9-500 mice were generated as a model of *C9orf72*-linked FTD/ALS by introducing a human full-length *C9orf72* gene harboring ~500 repeats of the GGGGCC sequence [34]. The mice reportedly produce RNA foci, DPR and TDP-43 inclusions, and neurodegeneration in the brain and spinal cord, showing motor dysfunction at 4 months and decreased survival between 5 and 20 months in acute progressive individuals. We noticed that the mice bred in our animal facility showed cognitive impairment at 4.5 months, but their motor function remained normal even at 12 months, suggesting that our mice belong to a slowly progressive type [34]. Intranasal rifampicin treatment for 1 month prevented *C9orf72* HRE-related neuropathologies and improved the memory of C9-500 mice at 5.5 to 6 months. In addition, phosphorylation of PKR was significantly attenuated in treated mice, suggesting that rifampicin suppressed RAN translation. Thus, our findings suggest a therapeutic potential of nasal rifampicin in the prevention of *C9orf72*-linked disorders.

## 2. Materials and Methods

### 2.1. Mice

FVB/NJ-Tg(C9orf72)500Lpwr/J mice, also known as C9-500 mice, were purchased from the Jackson Laboratory (Bar Harbor, ME, USA). This mouse line was generated as a model of *C9orf72*-linked FTD/ALS that expresses human *C9orf72* gene with ~500 hexanucleotide (GGGGCC) repeats using the bacterial artificial chromosome (BAC) vector [34]. The transgenic (Tg) mice were mated with wild-type FVB/NJc1 mice and maintained as heterozygotes for the transgene in our animal facility.

### 2.2. Behavioral Tests

Cognitive function of the mice was assessed at 4.5 months by the Morris water maze test, and motor function was examined at 4.5 and 12 months by the rotarod and inverted screen tests, as described previously [33].

### 2.3. Immunohistochemical Analysis

To study age-dependent neuropathology in the mice, brain sections of Tg and non-Tg littermates were prepared at ages 3, 6, and 12 months, as described previously [33]. For staining of pTDP-43, synaptophysin, and NeuN, sections were boiled in 10 mM of citrate buffer, pH6 for 30 min to expose the antigens. After blocking with 10% calf serum overnight, the sections were stained with antibodies to DNA/RNA G-quadruplex (BG4; Absolute antibody, Cleveland, UK), poly-GA, poly-GP, poly-GR (all from Cosmo Bio, Tokyo, Japan), pSer409/410-TDP-43 (Cosmo Bio), synaptophysin (SVP-38; Sigma-Aldrich, St. Luis, MO, USA), NeuN (Chemicon, Temecula, CA, USA), and Iba1 (Fujifilm-Wako, Osaka, Japan), essentially as described previously [33]. The staining was followed by a biotin-labeled second antibody (Vector Laboratories, Burlingame, CA, USA), horseradish peroxidase (HRP)-conjugated avidin-biotin complex (Vector Laboratories), and an HRP substrate, diaminobenzidine (DAB), or an FITC-labelled second antibody (Jackson Laboratory) only for synaptophysin. The stained specimens were viewed under a BZ-X800 fluorescence microscope (Keyence, Osaka, Japan), and the images of certain brain regions were photographed. Neuropathologies were evaluated by measuring the staining intensity or area or by counting positive puncta or cells in a constant area in each photograph using NIH ImageJ software (ImageJ bundled with 64-bit Java 1.8.0_172; https://imagej.nih.gov/ij/, accessed on 27 March 2022).

To examine the formation of nuclear and cytoplasmic inclusions, the sections were double stained with antibodies to G-quadruplex and hnRNP H1 (Proteintech, Rosemont, IL, USA) without pretreatment for RNA foci, and pSer409/410-TDP-43 and amyloidogenic protein oligomers (F11G3; Sigma-Aldrich) after pretreatment at pH6 for TDP-43 oligomer inclusions. For DPR inclusions, the sections were double stained with antibodies to poly-GA, poly-GP, poly-GR and SQSTM1/p62 (A-6; Santa Cruz Biotechnology, Dallas, TX, USA) without pretreatment. The staining was followed by FITC- and rhodamine-labelled secondary antibodies (Jackson Laboratory). The specimens were then treated with TrueBlack Plus Lipofuscin (Biotium, Fremont, CA, USA) to quench the autofluorescence and mounted with Vectashield vibrance antifade mounting medium with DAPI (Vector Laboratories). Images of the stained sections were taken under a BZ-X800 fluorescence microscope, and cells having inclusions were counted in a constant area.

### 2.4. Rifampicin Treatment

Four and a half to 5-month-old C9-500 mice were divided into two groups. One group was treated with rifampicin and the other with carboxymethylcellulose (CMC) every day from Monday to Friday for 4 weeks. Rifampicin (Sigma-Aldrich) was dissolved to 10 mg/mL in 0.5% low-viscosity CMC (Sigma-Aldrich). Ten microliters of rifampicin (i.e., 0.1 mg) or CMC solution was administered intranasally, as described previously [33]. Non-Tg littermates were treated with CMC alone. Following the 1-month treatment, cognitive function of the mice was tested by the water maze test, during which rifampicin treatment was continued. After the behavioral test, each group was divided into two groups: one group for immunohistochemical analysis and the other for future biochemical analysis. For immunohistochemistry, brain sections were prepared as described above, while for biochemical analysis, whole brains were removed and frozen at −80 °C until use.

### 2.5. Gel Shift Assay for G-Quadruplex

The gel shift assay to detect G-quadruplex formation was performed essentially as described previously [8]. Sense (GGGGCC)_4_, antisense (GGCCCC)_4_, and control (ATGC)_6_ oligonucleotides were synthesized and solubilized to 100 μM in 0.89 M of Tris-borate buffer, pH8.3 containing 0.02 M of EDTA (TBE). KCl was solubilized to 400 mM in TBE, and rifampicin was solubilized to 100 mM in DMSO. Five microliters of DNA solution, 25 μL of KCL or TBE alone, and 1 μL of rifampicin or DMSO alone were mixed with 69 μL of TBE to make the final concentrations 5 μM of DNA, 100 mM of KCl, and 1 mM of rifampicin. The mixtures were incubated at 98 °C for 3 min and cooled down to room temperature slowly. The samples were mixed with Blue/Orange Loading Dye (Promega, Madison, WI, USA) and subjected to native PAGE with 20% polyacrylamide gel (Novex TBE Gels, Invitrogen, Carlsbad, CA, USA) and TBE running buffer containing 50 mM KCl. The gels were stained with SYBR Gold nucleic acid gel stain (Invitrogen) for 10 min, and images were obtained using an ImageQuant LAS 500 image analyzer (GE Healthcare, Hino, Japan).

### 2.6. Immunohistochemistry for Phosphorylated PKR

To test the possibility that rifampicin affects RAN translation, the levels of phosphorylated PKR, a regulator of RAN translation [31], were examined by immunohistochemistry. Brain sections were boiled in 10 mM of citrate buffer, pH6 for 30 min to expose the antigens. After blocking, the sections were stained with an anti-phospho-PKR (Thr446) antibody (MilliporeSigma, Burlington, MA, USA) followed by a biotin-labeled secondary antibody, HRP-conjugated avidin-biotin complex, and DAB. The stained specimens were viewed under a BZ-X800 fluorescence microscope, and the levels of phospho-PKR were measured by quantifying the staining intensity in a constant area using NIH ImageJ software.

### 2.7. Statistical Analysis

All experiments and data analyses were performed under unblinded conditions. A comparison of means among more than two groups was performed by ANOVA or two-factor repeated measures of ANOVA (for the Morris water maze test) followed by Fisher’s PLSD test. Differences with a *p*-value of < 0.05 were considered significant.

## 3. Results

**Figure 1:**
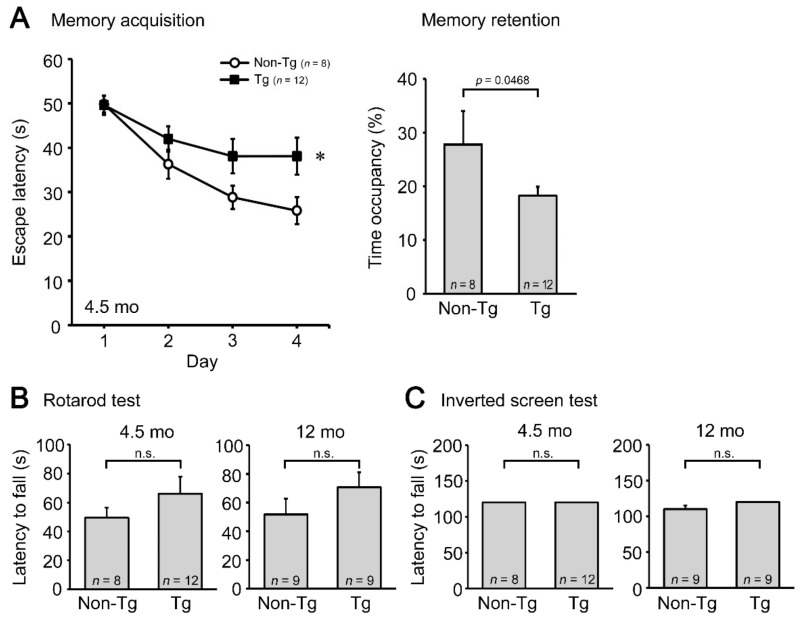
Cognitive and motor functions of C9-500 mice. Tg and non-Tg littermates were subjected to the Morris water maze (**A**), rotarod (**B**), and inverted screen tests (**C**) at 4.5 and 12 months (mo). Tg mice showed impaired cognitive function in both memory acquisition and retention (probe trial) tests at 4.5 months, but no motor deficits even at 12 months. * *p* = 0.0497 vs. non-Tg littermates. The number of mice analyzed was *n* = 12 (5 male and 7 female) for 4.5-month-old Tg mice, *n* = 8 (4 male and 4 female) for age-matched non-Tg littermates, *n* = 9 (7 male and 2 female) for 12-month-old Tg mice, and *n* = 9 (5 male and 4 female) for age-matched non-Tg littermates.

Initially, we studied the pathological phenotypes of C9-500 mice bred in our animal facility. Cognitive function of the mice was assessed at 4.5 months by the Morris water maze test, and motor function was examined at 4.5 and 12 months by the rotarod and inverted screen tests. Compared with non-Tg littermates, Tg mice showed significantly impaired memory but no motor deficit at 4.5 months (Figure 1). Motor function of the Tg mice remained normal even at 12 months. These results indicate that cognitive dysfunction precedes motor deficit in these mice and that the mice can be used as a model of FTD at least between 4.5 and 12 months. According to the founder’s criteria [34], these mice are considered to be a slowly progressive type.

**Figure 2:**
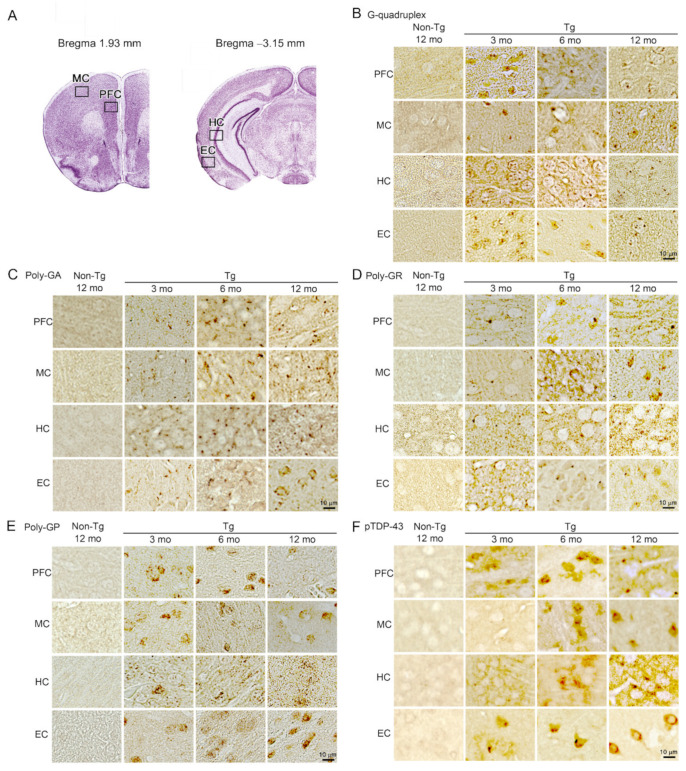
C9orf72 HRE-related pathologies in C9-500 mice. (**A**) Brain sections showing the prefrontal cortex (PFC), motor cortex (MC), hippocampus (HC), and entorhinal cortex (EC) were prepared at 3, 6, and 12 months. The sections were stained for G-quadruplex (**B**), poly-GA (**C**), poly-GR (**D**), poly-GP (**E**), and pTDP-43 (**F**). G-quadruplex and DPR accumulation were detected at 3 months in all regions. pTDP-43 accumulation appeared in the PFC and EC at 3 months and in the MC and HC at 6 months. The number of mice analyzed was *n* = 3 (2 male and 1 female) for each group. These age-dependent pathologies in each brain region were observed in all mice tested.

**Figure 3:**
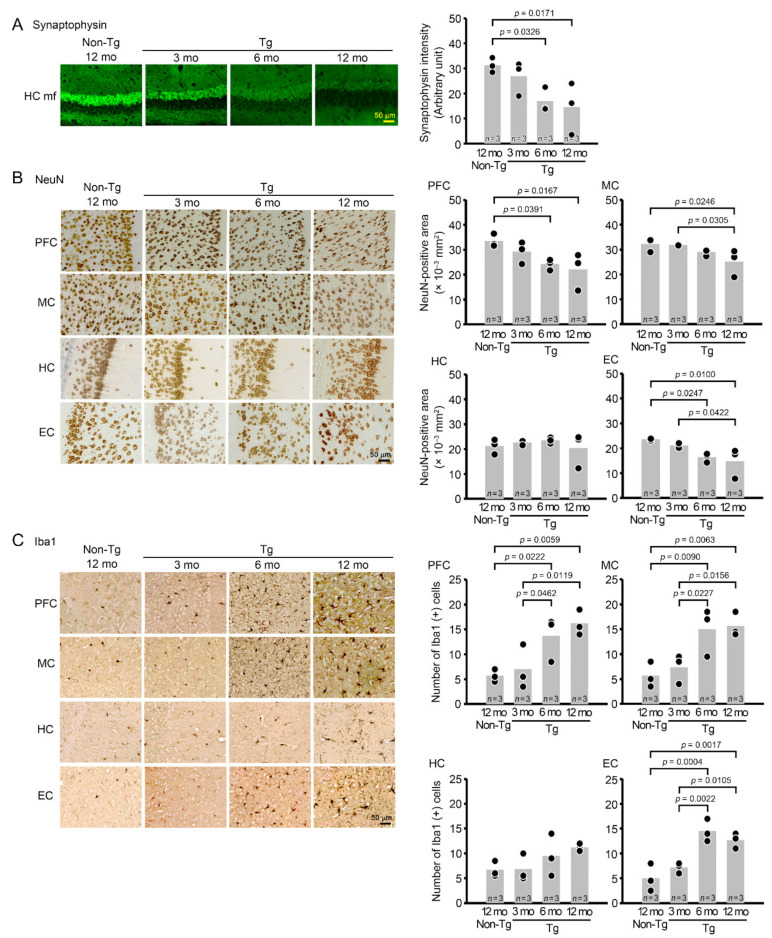
Synapse loss, neuronal loss, and microglial activation in C9-500 mice. Brain sections were stained for synaptophysin (**A**), NeuN (**B**), and Iba1 (**C**). Synaptophysin intensity was measured in a constant area (50 × 50 μm) of the hippocampal mossy fibers (HC mf). The levels began to decrease at 6 months. The NeuN-positive area and Iba1-positive cells were quantified in a constant area (280 × 370 μm) of the PFC, MC, HC, and EC. Neuronal loss was detected at 6 months in the PFC and EC and at 12 months in the MC but not in the HC. Microglial activation appeared at 6 months in the PFC, MC, and EC, but not in the HC even at 12 months. The number of mice analyzed was *n* = 3 (2 male and 1 female) for each group.

Then, we studied neuropathology of the mice by immunohistochemistry. Brain sections were prepared at 3, 6, and 12 months and stained with antibodies for DNA/RNA G-quadruplex, poly-GA, poly-GR, poly-GP, and pTDP-43. We examined the prefrontal cortex (PFC), motor cortex (MC), hippocampus (HC), and entorhinal cortex (EC) (Figure 2A), which are vulnerable regions in FTD and ALS. G-quadruplex appeared at 3 months in all brain regions we tested (Figure 2B). The three DPRs also started to accumulate at 3 months in all brain regions (Figure 2C–E). pTDP-43 was detected at 3 months in the PFC and EC and at 6 months in the MC and HC (Figure 2F). Then, brain sections were stained with antibodies for synaptophysin, the neuronal marker NeuN, and the microglial marker Iba1. Synaptophysin levels were measured in hippocampal mossy fibers. Significant synapse loss was detected in Tg mice at 6 months (Figure 3A). NeuN-positive areas and Iba1-positive cells were quantified in the PFC, MC, HC, and EC. Significant neuronal loss appeared in the PFC and EC at 6 months and in the MC at 12 months but barely in the HC even at 12 months (Figure 3B). In addition, significant microglial activation was observed at 6 months in the PFC, MC, and EC, as was a similar but not significant trend in the HC (Figure 3C). These results indicate that G-quadruplex formation and DPR accumulation were the initial pathology appearing in C9-500 mice followed by pTDP-43 accumulation. Furthermore, neurodegeneration was associated with the accumulation of pTDP-43, and the most susceptible brain regions in C9-500 mice were the PFC and EC. These observations appear to support our notion that mice prior to 12 months meet the requirements for a model of FTD.

**Figure 4:**
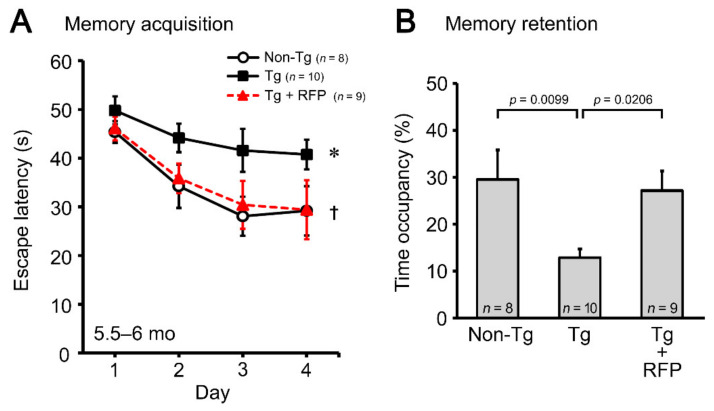
Effect of rifampicin on the cognition of C9-500 mice. Intranasal rifampicin (RFP) treatment at 0.1 mg/day for 1 month significantly improved cognitive function in both the memory acquisition (**A**) and retention (**B**) tests in 5.5- to 6-month-old Tg mice. * *p* = 0.0155 between Tg and non-Tg groups, ^†^ *p* = 0.0271 between Tg + RFP and Tg groups. The number of mice analyzed was *n* = 9 (6 male and 3 female) for rifampicin-treated Tg mice, *n* = 10 (6 male and 4 female) for CMC-treated Tg mice, and *n* = 8 (5 male and 3 female) for non-Tg littermates.

Thus, we decided to use 4.5- to 5-month-old mice to investigate the therapeutic potential of rifampicin against *C9orf72*-linked FTD. Tg mice were divided into two groups. One group was treated with intranasal rifampicin at 0.1 mg/day for 1 month and the other with CMC alone. Non-Tg littermates were treated with CMC. Following treatment, cognitive function of the mice was assessed by the water maze test. Intranasal rifampicin significantly improved the memory of Tg mice to a level similar to non-Tg littermates (Figure 4).

**Figure 5:**
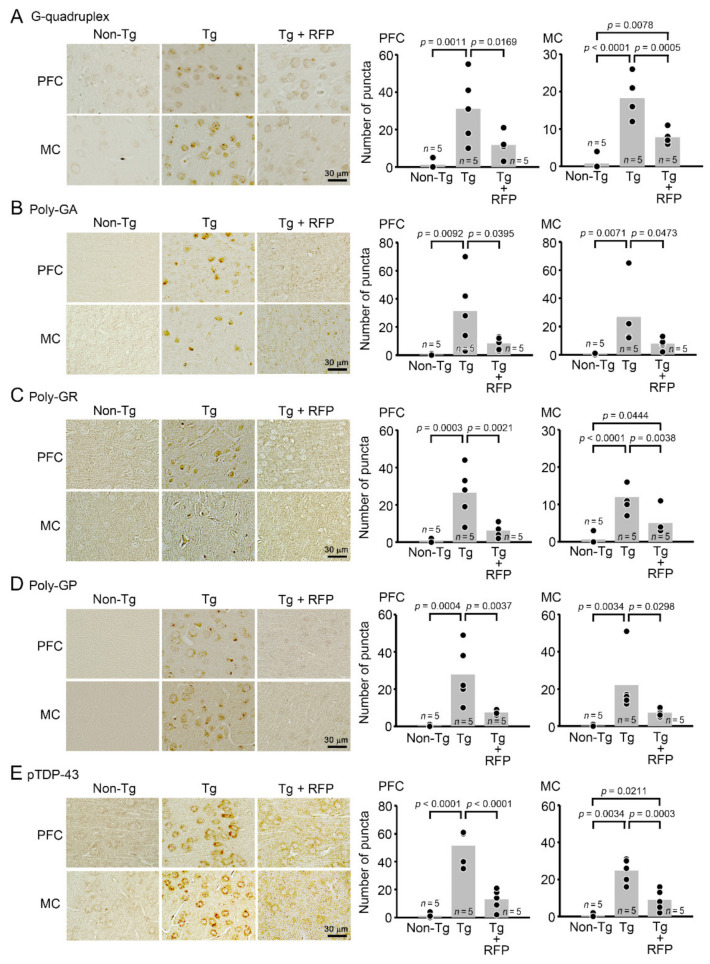
Effects of rifampicin on HRE-related pathologies in C9-500 mice. After the behavioral test, brain sections were prepared and stained with antibodies for G-quadruplex (**A**), poly-GA (**B**), poly-GR (**C**), poly-GP (**D**), and pTDP-43 (**E**). These pathologies were evaluated in the PFC and MC by counting immuno-positive puncta in a constant area (280 × 370 μm). Intranasal rifampicin (RFP) treatment significantly reduced the levels of G-quadruplex, DPR, and pTDP-43 puncta. The number of mice analyzed was *n* = 5 (3 male and 2 female) for each group.

**Figure 6:**
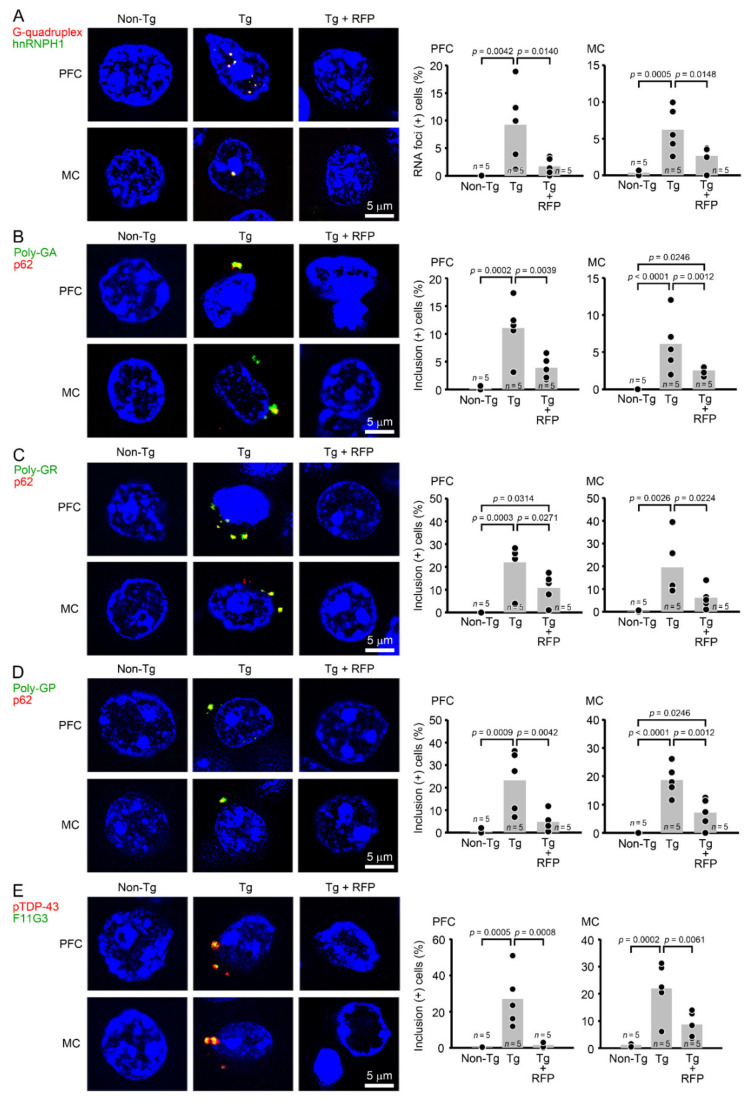
Effects of rifampicin on RNA foci and cytoplasmic inclusions in C9-500 mice. Brain sections were stained with antibody combinations to G-quadruplex and hnRNP H1 for RNA foci (**A**), DPRs and p62 for DPR inclusions (**B**–**D**), and pTDP-43 and amyloidogenic protein oligomers (F11G3) for TDP-43 oligomer inclusions (**E**) with a nuclear dye, DAPI. These pathologies were evaluated in the PFC and MC by counting cells having double-positive puncta (green + red = yellow) versus total cells (blue DAPI-positive) in a constant area (280 × 370 μm). Tg mice showed the formation of RNA foci and DPR and TDP-43 inclusions. Intranasal rifampicin (RFP) treatment significantly attenuated these pathologies.

**Figure 7:**
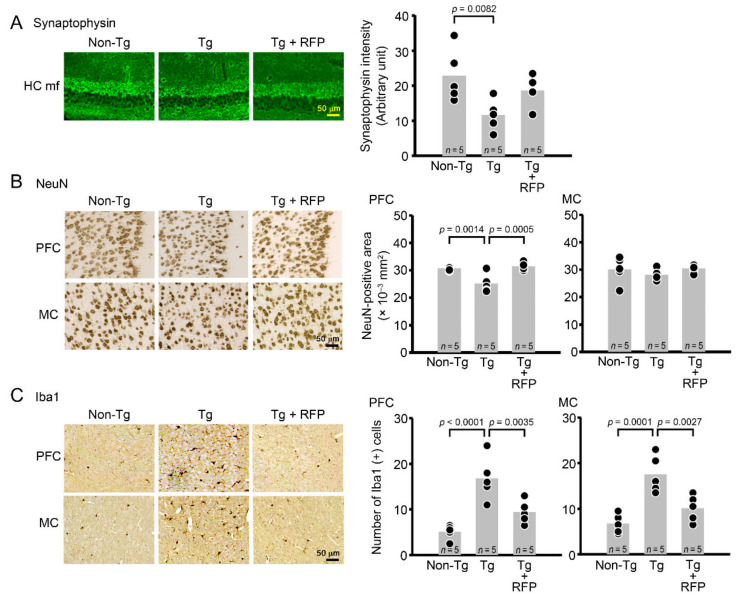
Effects of rifampicin against synapse loss, neuronal loss, and microglial activation in C9-500 mice. Brain sections were stained with antibodies for synaptophysin (**A**), NeuN (**B**), and Iba1 (**C**). Synaptophysin intensity was measured in a constant area (50 × 50 μm) of the hippocampal mossy fibers (HC mf), whereas NeuN-positive area and Iba1-positive cells were quantified in a constant area (280 × 370 μm) of the PFC and MC. Intranasal rifampicin (RFP) treatment significantly rescued synapse loss in the HC, neuronal loss in the PFC, and microglial activation in the PFC and MC.

After the behavioral test, each group was divided into two groups: one for immunohistochemical analysis and the other for future biochemical analysis. In the immunohistochemistry, brain sections were prepared and stained with antibodies for G-quadruplex, poly-GA, poly-GR, poly-GP, and pTDP-43. We evaluated these pathologies in the PFC and MC. Intranasal rifampicin significantly reduced the levels of G-quadruplex, DPR, and pTDP-43 puncta (Figure 5). We further studied whether these pathological molecules constituted RNA foci and cytoplasmic inclusions. RNA foci are dominantly formed in the nucleus and occasionally in the cytoplasm and composed of RNA G-quadruplex and RNA-binding proteins, such as hnRNP H. DPR inclusions are usually formed in the cytoplasm and occasionally in the nucleus and involve autophagy- and ubiquitin-proteasome-related p62 protein. TDP-43 inclusions are formed in the cytoplasm and positive for aggregated pTDP-43. Thus, we stained brain sections with antibody combinations to G-quadruplex and hnRNP H for RNA foci, DPRs and p62 for DPR inclusions, and pTDP-43 and amyloidogenic protein oligomers (F11G3) for TDP-43 oligomer inclusions. Tg mice showed the formation of RNA foci and DPR and TDP-43 cytoplasmic inclusions in the PFC and MC (Figure 6). Intranasal rifampicin significantly attenuated these pathologies. Then, brain sections were stained with antibodies for synaptophysin, NeuN, and Iba1. Intranasal rifampicin significantly rescued synapse loss in hippocampal mossy fibers, neuronal loss in the PFC, and microglial activation in the PFC and MC (Figure 7). These results collectively suggest a therapeutic potential of nasal rifampicin in the prevention of *C9orf72*-linked disorders.

**Figure 8:**
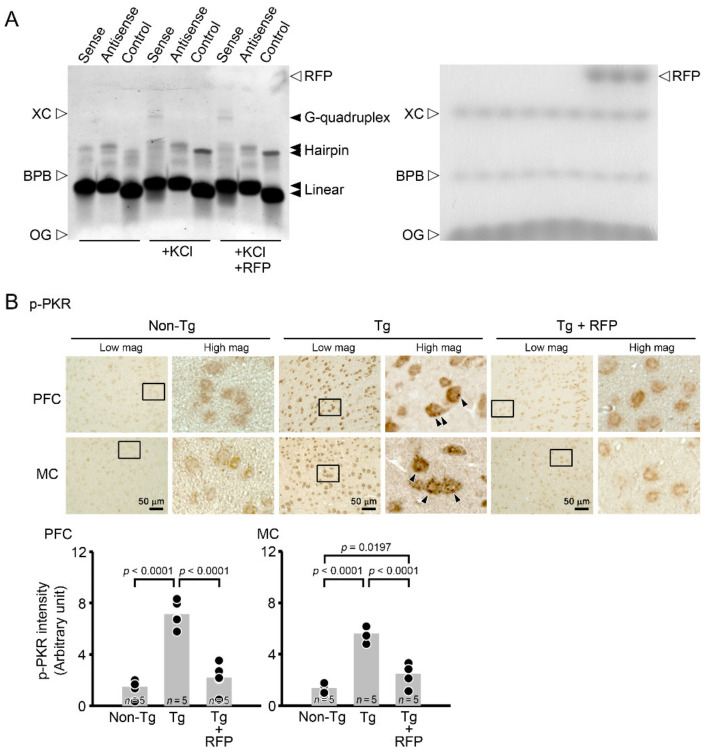
Effects of rifampicin on G-quadruplex formation and PKR phosphorylation. (**A**) In vitro effect of rifampicin on DNA G-quadruplex formation. Left and right photos show fluorescence and colorimetric images, respectively, of the same gel. Sense (GGGGCC)_4_ oligonucleotides formed G-quadruplexes in the presence of 100 mM of KCl, whereas antisense (GGCCCC)_4_ or control (ATGC)_6_ oligonucleotides did not. The addition of 1 mM of rifampicin (RFP) did not affect these observations. XC, xylene cyanol FF; BPB, bromophenol blue; OG, Orange G. Apparent molecular sizes of XC and BPB in 20% native gel are 45 bp and 12 bp, respectively. (**B**) Effect of rifampicin on PKR phosphorylation in C9-500 mice. Brain sections were stained with an anti-phospho-PKR (Thr446) antibody. Phospho-PKR intensity was measured in a constant area (280 × 370 μm) of the PFC and MC. Compared with non-Tg littermates, Tg mice showed a marked increase in phospho-PKR level with dense punctate staining (arrowheads) in the PFC and MC. Intranasal rifampicin (RFP) treatment significantly reduced the levels of phospho-PKR.

We next investigated the mechanisms by which rifampicin attenuated *C9orf72* HRE-related pathologies. We speculated that rifampicin may interact with DNA/RNA to inhibit G-quadruplex formation. To test this hypothesis, we examined the effect of rifampicin in vitro on G-quadruplex formation by synthetic (GGGGCC)_4_ oligonucleotides. A gel shift assay revealed that the DNA formed G-quadruplex in the presence of 100 mM of KCl [8] and that the addition of 1 mM of rifampicin failed to inhibit this formation (Figure 8A). Then, we considered that rifampicin may suppress RAN translation by inhibiting PKR phosphorylation like metformin [31]. We examined the levels of phosphorylated PKR in mouse brains by immunohistochemistry. Compared with non-Tg littermates, Tg mice showed a marked increase in phosphorylated PKR level in the PFC and MC (Figure 8B). Intranasal rifampicin significantly attenuated the levels of phosphorylated PKR. These results suggest that rifampicin prevents brain pathologies by suppressing RAN translation via inhibition of PKR phosphorylation.

## 4. Discussion

The C9-500 mice used in the present study were originally generated as a model of *C9orf72*-linked FTD/ALS [34]. According to the founder, the mice are divided into two subsets: an acute, rapidly progressive type and a slowly progressive type. The acute progressive mice (which corresponds to ~30–35% of all female mice) developed extensive neuronal loss in layers II/III throughout the cortex, layer V of the MC, the HC, cerebellum, and spinal cord at 2–5 months and showed hindlimb gait abnormalities at 4 months and a dramatic decrease in survival between 5–20 months. In contrast, the slow progressive mice showed a loss of motor neurons in the spinal cord, focal neurodegeneration in the neocortex, milder degeneration in the cerebellum, and no overt degeneration in the HC at 18 months. Male mice did not show decreased survival even at 1 year, but a large percentage of them (~43–45%) developed phenotypes similar to those found in slow progressive female mice by 1 year. Sense and antisense RNA foci were detected in the MC, HC, cerebellum, and spinal cord as early as 2 months. Poly-GA aggregates were detected throughout the brain at 2 months, with the first appearance in the retrosplenial cortex, and increased with age and disease. Poly-GP aggregates were also detected in the neocortex and thalamus in acute end-stage mice. Finally, TDP-43 aggregates were detected in degenerating neurons throughout the brain, including the HC and layers II/III and V of the MC, in acute end-stage mice. On the other hand, some researchers have claimed that these model mice do not show any reproducible abnormalities in survival, motor function, or neurodegeneration up to 1 year [35]. In addition, the Jackson Laboratory, who is a breeder of the mice, stated that the mice do not develop significant phenotypes, including paralysis (https://www.jax.org/strain/029099, accessed on 27 March 2022). To these arguments, the authors of the original paper later rebutted that several laboratories, including themselves, confirmed decreased survival and neurological and behavioral deficits in the mice [36]. They speculated that the discrepancy could be attributed to the methodological differences between groups.

In the present study, we initially characterized the pathological phenotypes of C9-500 mice purchased from the Jackson Laboratory and bred in our animal facility. We noticed that the mice showed cognitive impairment at 4.5 months but no motor deficit even at 12 months. The lack of motor deficit may be because mice predominantly belonged to the slow progressive type and/or our methodology to evaluate motor function is less sensitive than that of Liu et al. [34]. Alternatively, differences in the rearing environment may have caused differences in the gut microbiome of mice, which could affect motor and cognitive functions [37,38]. However, we observed apparent neuropathologies, including RNA foci, DPR and TDP-43 inclusions, synapse loss, neuronal loss, and microglial activation, in the PFC, MC, HC, and EC regions in an age-dependent manner starting from 3 months. These results indicate that C9-500 mice can be regarded as a model of *C9orf72*-linked FTD, at least between 4.5 and 12 months, in our rearing environment.

Neurodegenerative diseases are believed to be generally caused by the gain-of-toxic function of amyloidogenic proteins [39]. These proteins have been shown to become neurotoxic when they aggregate into oligomers [40]. For example, AD is initiated by the formation of synaptotoxic Aβ oligomers and proceeded by toxic aggregates of tau proteins. FTD is elicited by the oligomerization of tau or TDP-43, and TDP-43 inclusions are a hallmark in most cases of ALS. DLB and Parkinson’s disease are associated with α-synuclein oligomers. Meanwhile, *C9orf72* HRE mutation has been suggested to cause both gain-of-toxic function and loss-of-function [3,4]. *C9orf72* HRE produces RNA foci and DPR and TDP-43 inclusions, all of which possess cytotoxic effects [2,3,4]. At the same time, this mutation negatively affects the expression of C9orf72 by forming G-quadruplex structures in a promotor region, leading to haploinsufficiency of the protein [3,4,5]. Furthermore, RNA foci sequester some RNA-binding proteins [9,10,11], which may also result in loss-of-function of these proteins. In addition, HRE-derived RNAs and DPRs promote cytoplasmic LLPS and stress granule formation, in which TDP-43 tends to self-aggregate to form cytoplasmic inclusions [22,28]. This may cause the depletion of nuclear TDP-43, leading to loss-of-function of the protein.

**Figure 9:**
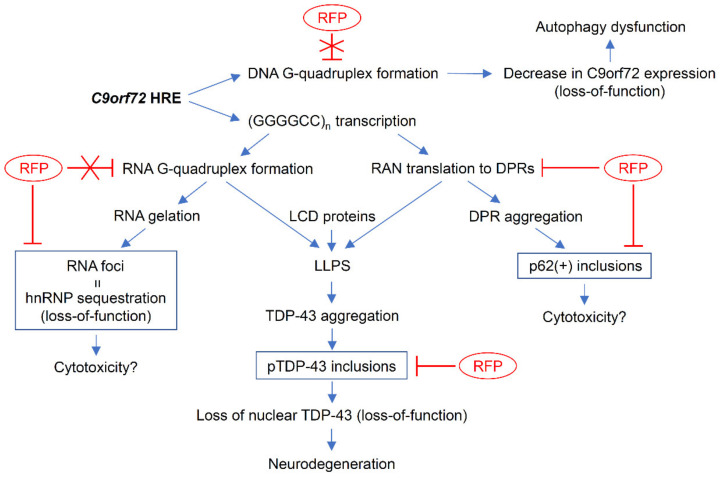
Pathways of *C9orf72* HRE-induced cytotoxicity and validated actions of rifampicin. *C9orf72* HRE mutation is presumed to cause disease by the loss-of-function and gain-of-toxic function of relevant proteins. HRE inhibits the transcription of *C9orf72* gene by forming the G-quadruplex structure in a promoter region, which causes haploinsufficiency of C9orf72 protein and resultant autophagy dysfunction. HRE also produces aberrant sense and antisense transcripts which form the G-quadruplex and hairpin structures and sequester RNA-binding proteins into RNA foci. This sequestering may cause disturbed RNA processing, leading to the RNA-mediated cytotoxicity. The HRE-derived sense and antisense RNAs generate five DPRs by RAN translation. These DPRs self-aggregate to form inclusions that involve an autophagy- and ubiquitin-proteasome-related protein, p62. In addition, HRE-derived RNAs and DPRs promote cytoplasmic LLPS to form stress granules, in which RNA, RNA-binding proteins with LCD, such as TDP-43, and translation machinery are condensed. These alterations accelerate the formation of cytoplasmic TDP-43 inclusions and the depletion of nuclear TDP-43, which causes loss-of-function of the protein. Rifampicin (RFP) does not inhibit the G-quadruplex formation (T-shaped red lines with X) but suppresses RAN translation by inhibiting PKR phosphorylation, preventing the formation of DPR and TDP-43 inclusions (T-shaped red lines). Rifampicin also attenuates RNA foci formation by unidentified mechanism.

We showed here that intranasal rifampicin prevented the formation of RNA foci and DPR and TDP-43 inclusions in C9-500 mice. These results suggest that rifampicin prevents not only the gain-of-toxic function but also loss-of-function caused by *C9orf72* HRE mutation, at least in part. Regarding the mechanism by which rifampicin attenuated the pathologies, there are several action points considered (Figure 9). We initially speculated that rifampicin may interact with DNA/RNA to inhibit G-quadruplex formation. However, rifampicin failed to inhibit this formation. Then, we considered that rifampicin may suppress RAN translation by inhibiting PKR phosphorylation like metformin [31]. Immunohistochemistry revealed that rifampicin treatment significantly decreased phosphorylated PKR levels in mouse brains, supporting our hypothesis. This explains why rifampicin was effective at reducing DPR and TDP-43 inclusions, but it remains unclear how rifampicin prevented RNA foci formation. *C9orf72* HRE-derived RNAs are shown to form multivalent base-pairing, which causes RNA gelation without requiring protein components and leads to RNA foci formation [41]. Notably, the RNA gelation and RNA foci formation can be inhibited in vitro by monovalent cations, such as ammonium acetate [41]. Thus, it may be that rifampicin prevented RNA foci formation by interfering with RNA base-pairing like monovalent cations. In neurodegenerative diseases, aggregated protein pathologies are presumed to propagate in the brain by the mechanism of cell-to-cell transmission [42]. DPRs and TDP-43 have also been suggested to be transmitted intercellularly [43,44]. Previously, we demonstrated that intranasal rifampicin inhibits tau oligomer propagation in model mice [45]. Rifampicin may have attenuated the neuropathology in C9-500 mice by inhibiting the cell-to-cell transmission of DPRs and TDP-43. In the present study, we treated C9-500 mice with rifampicin at 0.1 mg/day for 1 month. Although nasal administration of rifampicin is safer than oral administration [46], long-term treatment with rifampicin at this dose may cause adverse effects, such as liver dysfunction and drug-drug interaction. However, we previously showed that when rifampicin was administered together with resveratrol, the toxicity of rifampicin was completely eliminated in mice [47]. The therapeutic effects of this combinatorial medicine against *C9orf72*-linked disorders remain to be studied.

It is known that the expansion of short nucleotide repeats in both coding and non-coding regions underlies more than fifty human disorders [48,49]. Although the responsible genes and pathological phenotypes are different for each disease, the pathological mechanism is likely common among them. For example, in Huntington’s disease (HD), spinocerebellar ataxia (SCA), dentatorubral-pallidoluysian atrophy, and spinal bulbar muscular atrophy, CAG repeats in coding regions expand to more than 20–300 times and produce a prolonged poly-Q sequence in translated proteins which then tend to self-aggregate to form inclusions and exhibit cytotoxicity [50]. In contrast, myotonic dystrophy (DM), benign adult familial myoclonic epilepsy, fragile X syndrome, fragile X-associated tremor/ataxia syndrome, several types of SCA, and other nucleotide repeat expansion disorders possess more than 30–10,000 times expansion of short nucleotide repeats (such as CTG, CCTG, TTTCA, etc.) in non-coding regions, which then generate abnormal RNAs and proteins and cause cytotoxicity via both gain-of-toxic function and loss-of-function, similar to *C9orf72*-linked FTD/ALS [51]. Recently, it has been demonstrated that RAN translation also occurs in HD and that, in addition to poly-Q, four novel RAN proteins, including poly-A, poly-S, poly-L, and poly-C, are synthesized from sense and antisense repeat RNAs to accumulate in the brains [52]. Furthermore, metformin has been shown to exhibit beneficial effects in model mice of HD and human patients with HD and DM type I [53,54,55,56]. Considering the commonality of their pathogenesis and rifampicin’s metformin-like action, rifampicin could be effective against these coding and non-coding repeat disorders.

In summary, intranasal rifampicin attenuated pathological phenotypes in C9-500 mice, including RNA foci, DPR and TDP-43 inclusions, neurodegeneration, and cognitive impairment. Taken together with our previous findings that nasal rifampicin has beneficial effects in model mice of AD, tau-associated FTD, and DLB, the present results suggest that rifampicin is a good candidate as a preventive medicine with a broad spectrum against neurodegenerative dementia. The effects of rifampicin on motor neuron diseases remain for future study.

## Data Availability

The data presented in this study are available upon request.
